# Cryoglobulinemic Vasculitis Masquerading as Bilateral Carpal Tunnel Syndrome

**DOI:** 10.7759/cureus.6423

**Published:** 2019-12-19

**Authors:** Oyintayo Ajiboye, Ishaan Vohra, Bashar Attar, Vatsala Katiyar, Benjamin Mba

**Affiliations:** 1 Internal Medicine, John H. Stroger Jr. Hospital of Cook County, Chicago, USA; 2 Internal Medicine, John H Stroger, Jr. Hospital of Cook County, Chicago, USA; 3 Gastroenterology and Hepatology, Rush University Medical Center, Chicago, USA; 4 Internal Medicine, John H Stroger Jr. Hospital of Cook County, Chicago, USA; 5 Internal Medicine, John H Stroger Jr Hospital of Cook County, Chicago, USA

**Keywords:** sensorimotor neuropathy, esrd, hepatitis c, mixed cryo, moneuritis multiplex, cryoglobulinemic vasculitis, diabetic neuropathy

## Abstract

Cryoglobulinemic vasculitis (CV) is a systemic inflammatory syndrome involving small- to medium-sized vessels. Almost half of hepatitis C-infected patients have detectable cryoglobulins levels, but only very few develop clinical manifestations. In this case report, we bring forth a diagnostic challenge of CV. A 52-year-old man with untreated hepatitis C (high viral load), diabetes mellitus, hypertension, and chronic kidney disease 4 (CKD) with solitary left kidney presented with one month of bilateral hand pain that started in his right hand, progressed to involve the left with numbness more on the palmer aspects and lateral three fingers. Physical exam was significant for bilateral positive Phalen and Tinel's sign. CV occurs due to precipitation of immune complexes within the vessels. Palpable purpura, arthralgia, and weakness have been described as the core symptoms of CV. However, progressive acute to subacute neuropathy, especially bilateral, should raise concerns for vasculitic neuropathy.

## Introduction

Cryoglobulinemic vasculitis (CV) is a systemic inflammatory syndrome that affects small- to medium-sized vessels involving the skin, joints, kidney, and peripheral nerves. It occurs due to the presence of cryoglobulins, which are circulating immunoglobulins that precipitate at temperatures below 37 °C and re-dissolve on rewarming [1-2]. While cryoglobulins can be found in serum at detectable levels without clinical manifestations, clinically significant cryoglobulinemia is uncommon and seen only in one per 100,000 people. In all, 65% of hepatitis C-infected individuals have detectable levels of circulating cryoglobulins, while active CV occurs in only 15% [2-4].

Peripheral neuropathy, one of the clinical features of CV, presents as pain, weakness, and numbness in the form of distal sensory or sensory-motor neuropathy [5]. The peroneal nerve is the most commonly affected nerve. Tibial, ulnar, and median nerves are rarely involved [6]. Multiple non-contiguous nerves can be affected in the form of a mononeuritis multiplex but are uncommon especially as an initial isolated presentation in CV [5-6]. 

We describe a case of hepatitis C-related CV presenting as vasculitic mononeuritis multiplex initially diagnosed as carpal tunnel syndrome. This case report emphasizes the need to consider vasculitic syndromes as an etiology for acute and subacute neuropathies.

## Case presentation

A 52-year-old man with untreated hepatitis C (Viral load 520388, Genotype 1b), type 2 diabetes, hypertension, and chronic kidney disease 4 (CKD) with solitary left kidney presented with one month of bilateral hand pain that started in his right hand, progressed to the left with numbness on the palmer aspects, and lateral three fingers. He had no rash, arthritis, fevers, weight loss, or trauma. The examination was significant for bilateral positive Phalen and Tinel's sign, hyperesthesia with weakness on finger extension, and flexion of the lateral three digits. The initial impression was bilateral carpal tunnel syndrome secondary to diabetes mellitus.

**Figure 1:**
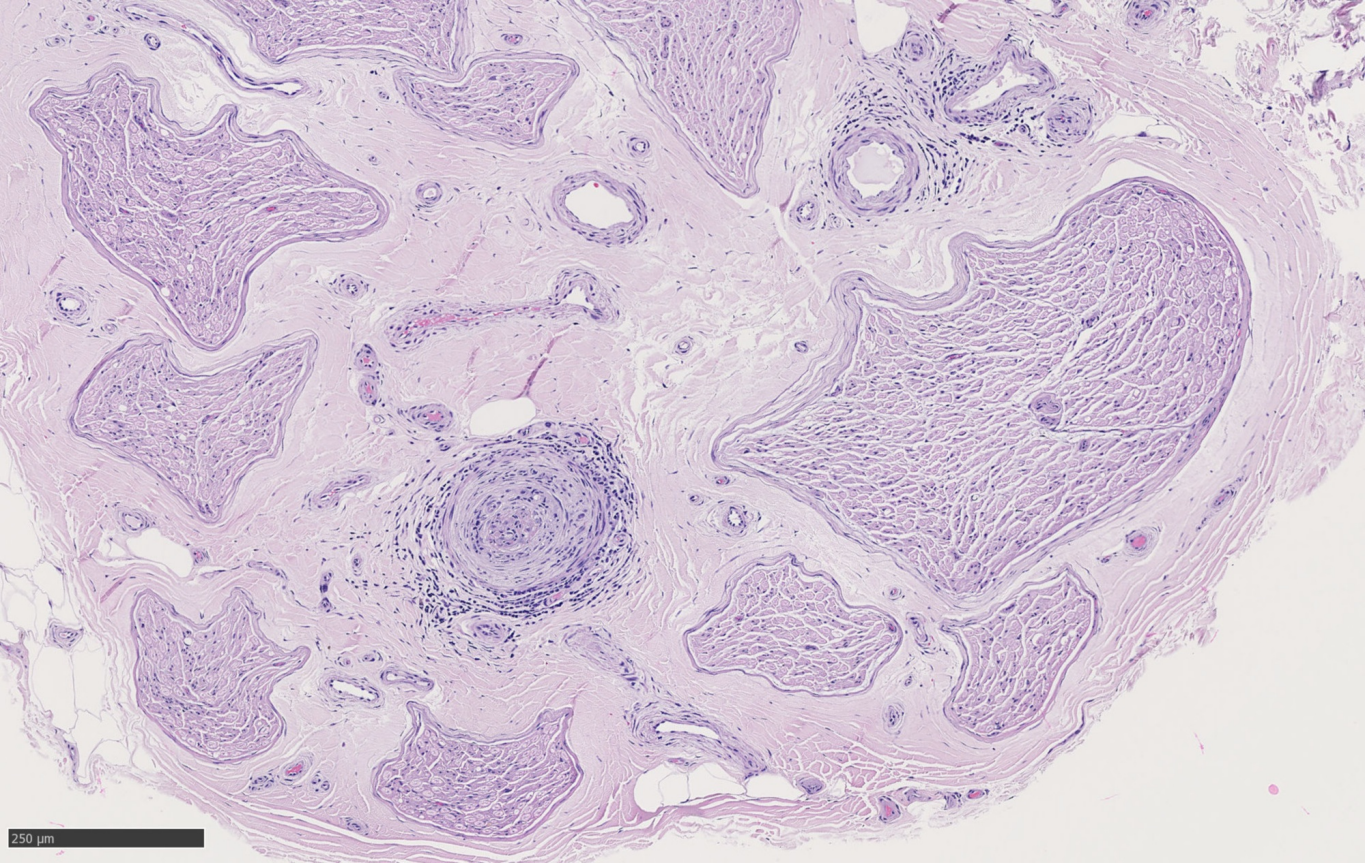
Cross-section of sural nerve with nerve bundles sampled healthy on HE_10x_05

**Figure 2:**
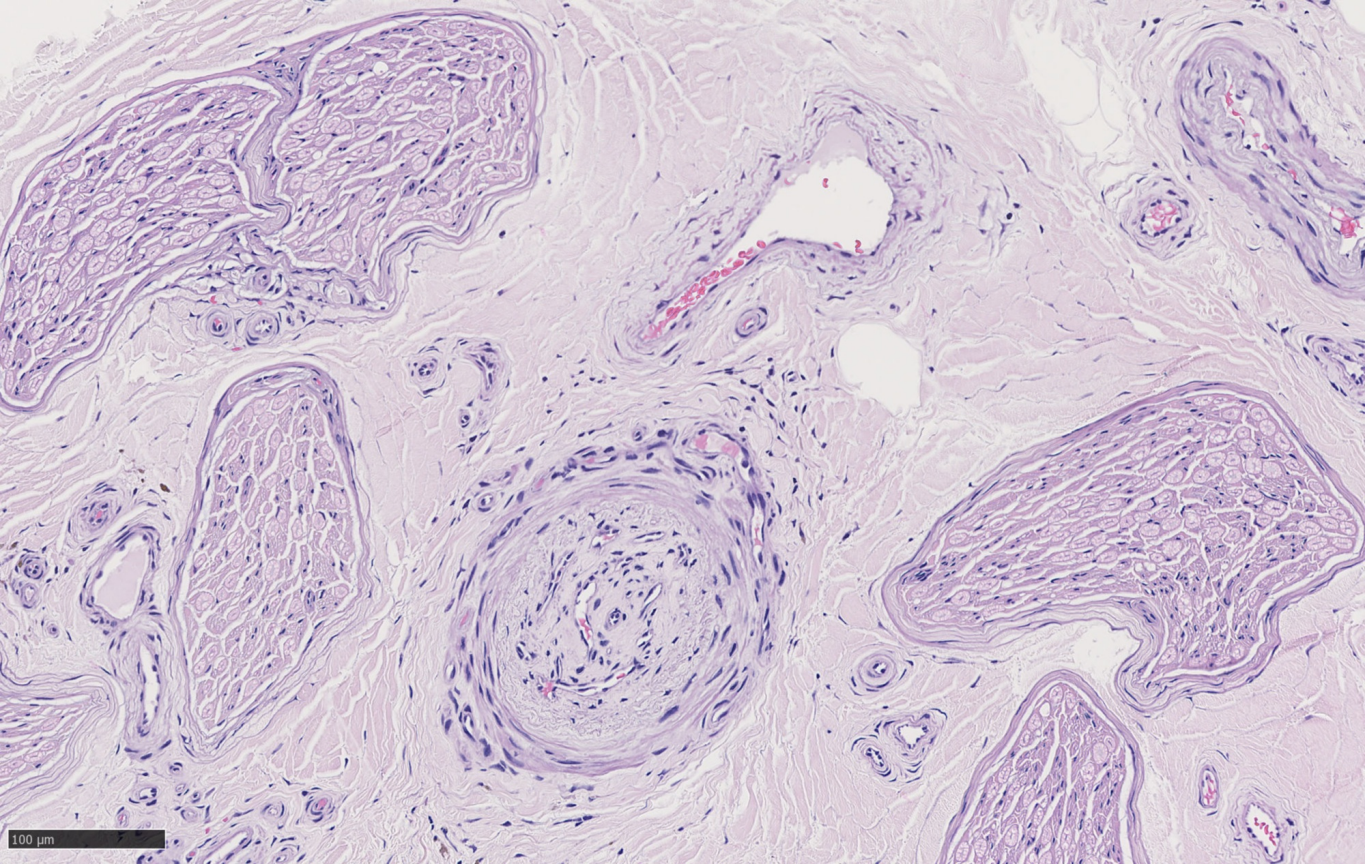
Cross-section of sural nerve with neovascularization best seen on HE_20x_03

**Figure 3:**
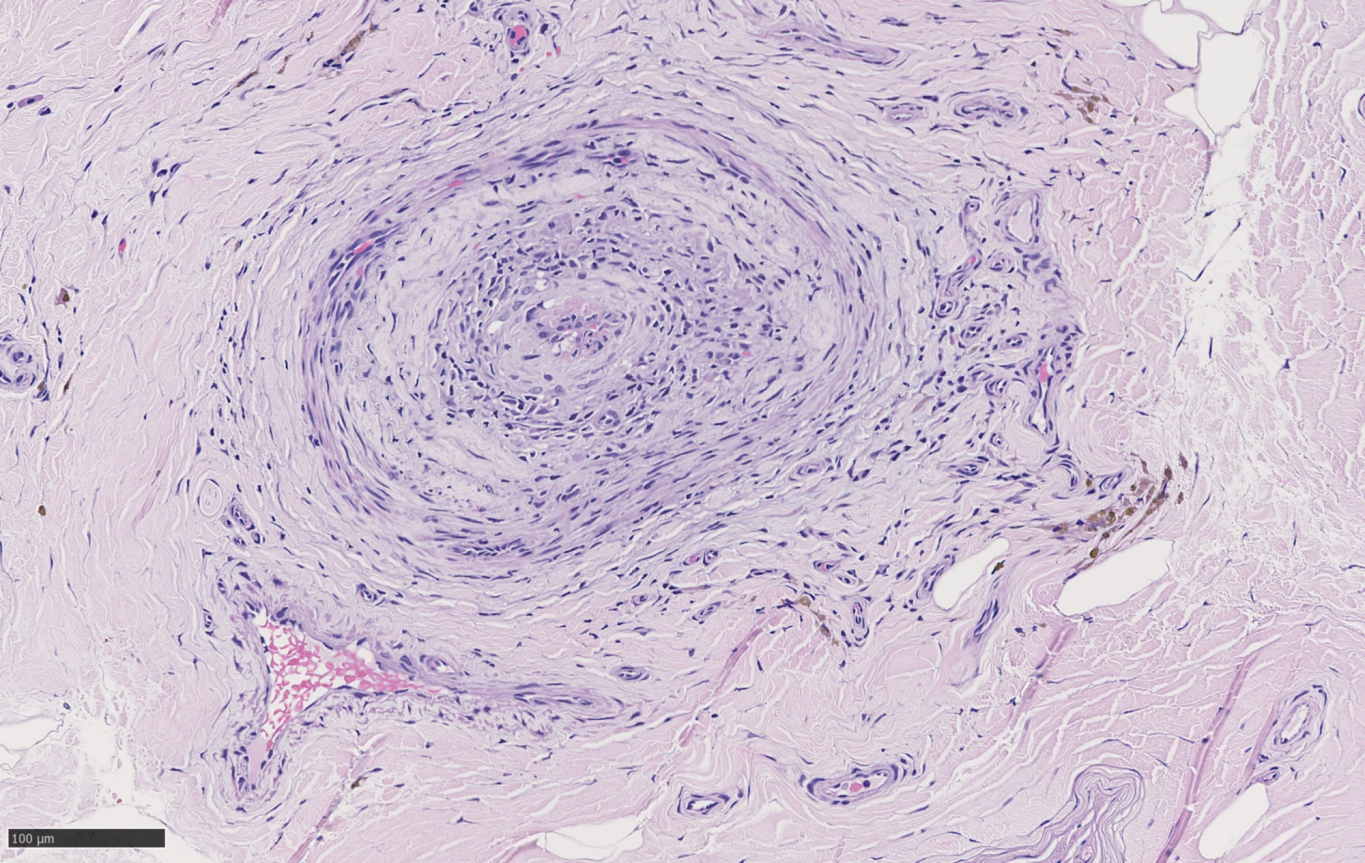
Cross-section of sural nerve with fibrinoid necrosis and hemosiderin deposition best visible on HE_20x_02

**Figure 4:**
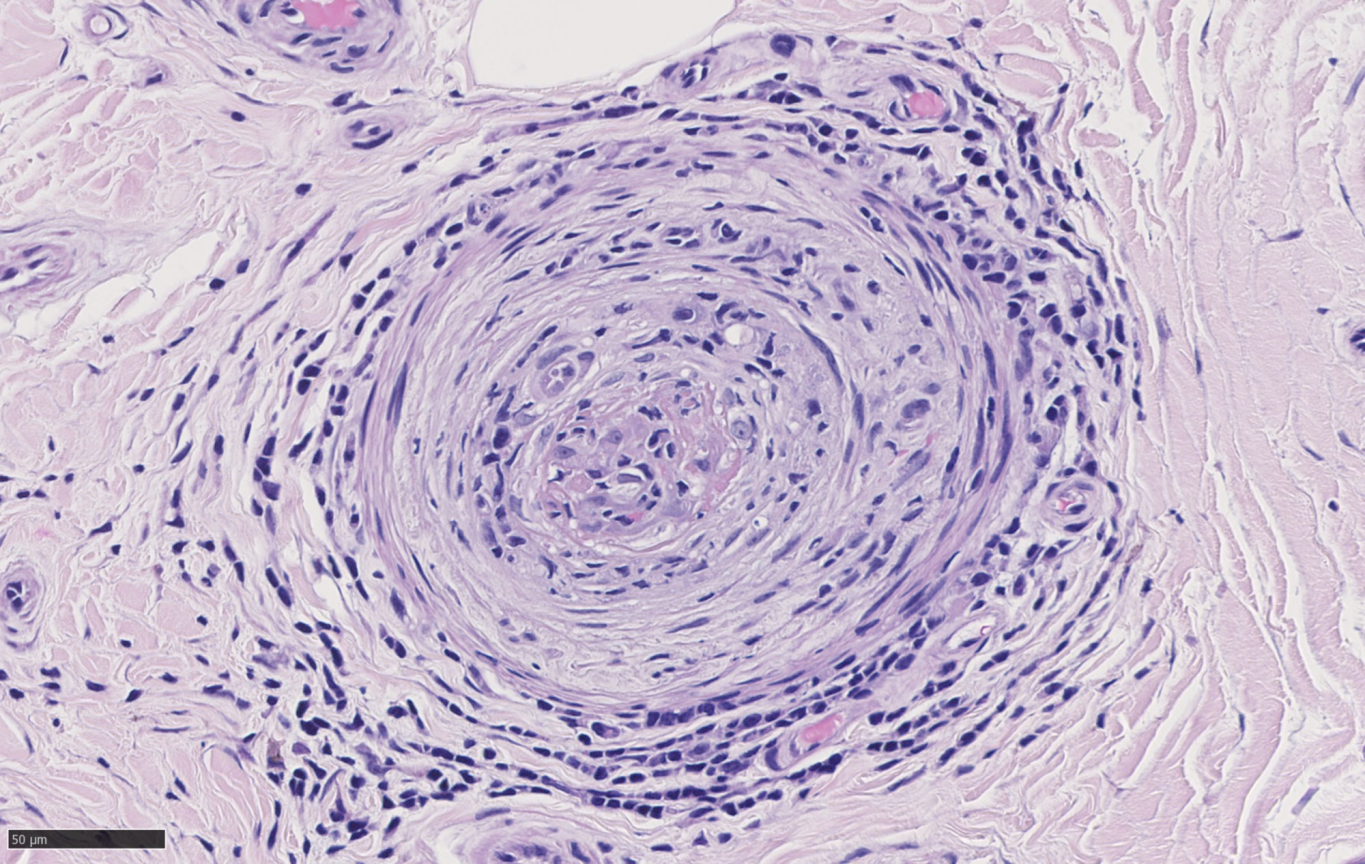
Cross-section of sural nerve with fibrinoid necrosis plus the perivascular mononuclear inflammation best seen on HE_40x_05

Investigations revealed positive serum cryoglobulins with low C3, C4, positive rheumatoid factor, negative cyclic citrullinated peptide (CCP), and nephrotic range proteinuria. Antinuclear antibodies (ANA), antineutrophil cytoplasmic antibodies (C-ANCA, myeloperoxidase [MPO]/proteinase 3 [PR3]), human immunodeficiency virus (HIV), and rapid plasma reagin (RPR) were negative, and vitamin B12 and TSH were normal. Nerve conduction studies (NCS) showed non-length-dependent axonal loss in the proximal median nerve, indicative of mononeuritis multiplex. NCS showed nerve conduction sensory abnormalities in the left leg. The patient had a sural nerve biopsy revealing inflammation and vasculopathic changes on the paraffin sections consistent with vasculitis (Figure 1-4). Findings were seen on the hematoxylin and eosin stains with fibrinoid necrosis in the vessels, suggestive of active vasculitis (Figure 4). He was diagnosed with a mononeuritis multiplex secondary to hepatitis C-related cryoglobulinemia. 

He was planned for immunosuppressive therapy with glucocorticoids and rituximab but tested positive for latent tuberculosis. He was discharged on RIPE therapy and switched to rifampicin in the outpatient clinic after acid-fast bacteria (AFB) cultures were negative.

Fifteen weeks later, he was admitted for acute gastroenteritis and acute bilateral foot drop. Investigations showed his CKD 4 had progressed to CKD 5 requiring dialysis during the admission; both the CKD progression and bilateral foot drop were thought to be related to CV. He was started on prednisone and planned for concurrent rituximab therapy and hepatitis C treatment, pending resolution of the gastroenteritis.

## Discussion

Cryoglobulins are classified into three types based on the Brouet classification criteria. This system is based on the association with clinical presentations and underlying etiology [7]. Type 1 cryoglobulins are monoclonal immunoglobulins associated with B-cell lineage malignancy; type 2 and 3 are mixed cryoglobulins, often associated with persistent infections including hepatitis B and C virus infections, HIV, lymphoproliferative diseases, and autoimmune diseases such as systemic lupus erythematosus [3,7-8]. Vasculitis occurs due to precipitation of monoclonal immunoglobulins in small- to medium-sized vessels or precipitation of immune complexes in the microcirculation [7]. 

The classic Meltzer’s triad of purpura, arthralgia, and weakness was first described in 1966; its components occur in approximately 90%, 80%, and 50% of patients with CV, respectively [7-9]. Renal involvement occurs in approximately 30% of patients, ranging from isolated sub-nephrotic proteinuria or hematuria to nephritic or nephrotic syndromes to acute or chronic renal failure. The heart, gastrointestinal tract, and lungs can also be affected [2,7]. 

Peripheral neuropathy that is seen more in mixed cryoglobulinemia presents as distal sensory or sensory-motor polyneuropathy [9]. Severe neuropathy such as mononeuropathy multiplex seen in our patient is an uncommon initial presentation in CV [5,9]. Vasculitic neuropathy should be suspected when a patient presents with bilateral acute to subacute neuropathy and should be distinguished from entrapment neuropathy: carpal tunnel syndrome which has a more chronic course [4,6]. NCS aids in identifying the pattern of involvement, the presence of subclinical nerve involvement, and the selection of appropriate nerves for biopsy. In CTS, conduction slowing is localized only to the region of the median nerve passing through the carpal tunnel sheath, while in vasculitic neuropathies, there is asymmetric axonal injury seen as low amplitudes in sensory nerve and compound muscle action potential with normal or slow conduction [6,10]. 

The presence of cryoglobulins and low complements is a hallmark for CV: this should be followed by immunochemical analysis to detect immunoglobulins involved [2]. Tissue biopsy is generally not required for diagnosis but provides histopathologic evidence of vasculitis and helps determine patients that may need immunosuppression. In mixed cryoglobulinemia with mild to moderate disease, the primary focus is the treatment of the underlying cause while in severe to life-threatening disease; immunosuppressive therapy is indicated [2,9,11]. In the hepatitis C virus (HCV)-related severe CV, disease control is recommended before antiviral therapy [11]. All patients should be tested for latent tuberculosis prior to initiating immunosuppressive therapy; if testing is positive, treatment for latent tuberculosis should be completed before initiating therapy [12]. HCV-associated CV has a poorer prognosis compared to type 1 and non-HCV-associated CV with a 10-year survival rate of 63%. The most common causes of death are CNS involvement, infections, and advanced liver disease [9].

## Conclusions

CV presents with nonspecific symptoms including bilateral neuropathy which may also be explained by other disease entities. Physicians should have a high index of suspicion for CV when patients present with acute to subacute neuropathy and should initiate treatment as early as possible to prevent progression.
